# Commentary: A (Mito)SNO storm to protect the heart

**DOI:** 10.1016/j.xjon.2021.08.022

**Published:** 2021-08-20

**Authors:** Adishesh K. Narahari, J. Hunter Mehaffey

**Affiliations:** Division of Cardiac Surgery, Department of Surgery, University of Virginia Health System, Charlottesville, Va

**Figure:**
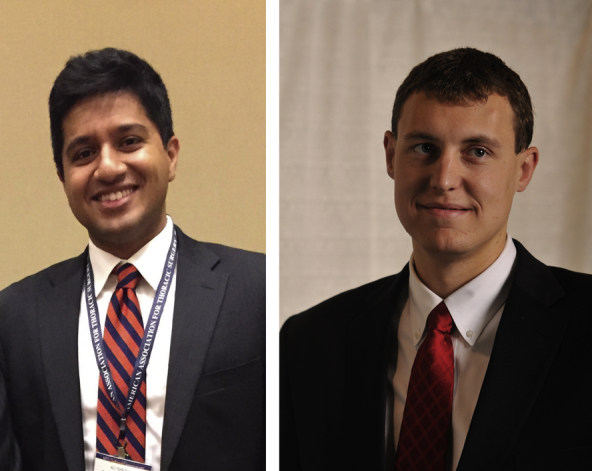
Adishesh K. Narahari, PhD (*left*), and J. Hunter Mehaffey, MD, MSc (*right*)

**Central Message**

MitoSNO, a mitochondrial-selective S-nitrosating agent, and diazoxide, a K_ATP_ channel agonist, are cardioprotective agents separately but not synergistically.

See Article page 338.

In this issue of the *Journal of Thoracic and Cardiovascular Surgery Open*, Ahmad and colleagues^1^ have dissected the use of 2 potential cardioprotective additives: mitochondria-selective S-nitrosating agent (MitoSNO) and diazoxide. Perioperative ischemia–reperfusion injury in cardiac surgery is a critical issue. Cardioprotection to minimize the damage to the heart during ischemic time as well as from reperfusion injury is crucial to cardiac function after weaning from cardiopulmonary bypass.^2^ Numerous methods are currently used including but not limited to cardiac hypothermia and cardioplegia.

It has been shown that MitoSNO provides cardioprotection by S-nitrosation of a cysteine switch on mitochondrial complex I. The S-nitrosation provides protection by limiting the entry of electrons from NADH into the respiratory chain, thereby limiting the damage of reactive oxygen species during the reperfusion phase of ischemia-reperfusion injury commonly seen in cardiac surgeries.^3^ Preventing the reactivation of mitochondrial complex-I through reversible S-nitrosation is a salient and discrete mechanism of cardioprotection. Diazoxide, the second compound used in this work, functions through the opening of mitochondrial K_ATP_ (mito K_ATP_) channels.^4^ Although the exact mechanism is unknown, it is predicted that opening of mito K_ATP_ channels decreases Ca^2+^ flux from mitochondria and prevents Ca^2+^-induced myocyte contractility.^5^ Other theories in how mito K_ATP_ activation is protective have also been proposed and are being actively studied.

In this manuscript, Ahmad and colleagues very clearly show that the addition of MitoSNO or diazoxide to hyperkalemic cardioplegia are protective in both an isolated heart Langendorff model of global ischemia and with a cellular isolated myocyte ischemia-reperfusion model. When both diazoxide and MitoSNO were combined, Ahmad and colleagues predicted a synergistic effect to provide additional cardioprotection. However, this was not the case, as the combination was actually detrimental to cardioprotection. Each compound alone provided increased cardioprotection compared with the combined application.

The clinical applications from these findings cannot be overstated. Advancing cardioplegia formulations for strategies aimed at improved cardioprotection are paramount to furthering our technical expertise in cardiac surgeries utilizing cardiopulmonary bypass. We applaud Ahmad and colleagues in this important pursuit, and their key findings in this manuscript lay the groundwork for clinical trials testing new variations of cardioplegia to now include MitoSNO in our arsenal of cardioprotective agents.
